# Recent advances in understanding evolution of the placenta: insights from transcriptomics

**DOI:** 10.12688/f1000research.13115.1

**Published:** 2018-01-19

**Authors:** Anthony M. Carter

**Affiliations:** 1 Cardiovascular and Renal Research, Institute of Molecular Medicine, University of Southern Denmark, J. B. Winsloews Vej 21, DK-5000 Odense, Denmark

**Keywords:** Placentation, evolution, mammals, yolk sac, chorioallantoic placenta

## Abstract

The mammalian placenta shows an extraordinary degree of variation in gross and fine structure, but this has been difficult to interpret in physiological terms. Transcriptomics offers a path to understanding how structure relates to function. This essay examines how studies of gene transcription can inform us about placental evolution in eutherian and marsupial mammals and more broadly about convergent evolution of viviparity and placentation in vertebrates. Thus far, the focus has been on the chorioallantoic placenta of eutherians at term, the reproductive strategies of eutherians and marsupials, and the decidual response of the uterus at implantation. Future work should address gene expression during early stages of placental development and endeavor to cover all major groups of mammals. Comparative studies across oviparous and viviparous vertebrates have centered on the chorioallantoic membrane and yolk sac. They point to the possibility of defining a set of genes that can be recruited to support commonalities in reproductive strategies. Further advances can be anticipated from single-cell transcriptomics if those techniques are applied to a range of placental structures and in species other than humans and mice.

## Introduction

The placenta and fetal membranes of eutherian mammals show great diversity in their gross appearance and internal structure ^1^. How these diverse placental types evolved has fascinated developmental biologists since the time of Hubrecht ^2^. The advent of molecular phylogenetics placed taxonomy on a surer footing, revealing hitherto unforeseen relationships between the mammalian orders and offering an improved framework to interpret placental evolution ^3^. This led to analyses that focused on placental shape, internal structure, and the extent of trophoblast invasion of the uterine wall ^4– 8^. There was agreement that the most recent common ancestor (MRCA) of eutherians likely had a placenta that was discoid in shape, labyrinthine in structure, and highly invasive (probably hemochorial). Less attention was given to the other fetal membranes and to yolk sac (choriovitelline) placentation. We found that the embryo of the MRCA would have been nourished at first by a yolk sac placenta and subsequently by a chorioallantoic placenta ^4, 9^. Moreover, in contrast to human and other haplorrhine primates, but like many other mammals, the MRCA would have had a large allantoic sac ^4, 9^. The mammalian tree continues to be refined. It is now established that colugos (order Dermoptera) are the sister group to primates ^10^, and this supports a scenario for the evolution of another unique feature of human placentation: villous trees with fetal capillaries immersed in an intervillous space containing the maternal blood ^11^. This pattern is shared only by apes and Old World monkeys ^12^, although something similar emerged through convergent evolution in armadillos and South American anteaters ^13^.

The diversity of placental structure seemingly implies variations in function. The evolutionary basis for this is explored elsewhere ^14^. Placenta-specific genes are few but sometimes have arisen by duplication of existing genes and acquisition of a new function by the duplicate. An interesting exception highlighted by current research is the capture of retroviral envelope genes and their expression during trophoblast syncytialization ^15^. However, most recent advances in our understanding of the evolution of placental function are the fruits of transcriptomics. They are afforded particular weight in this review.

Although the focus has been on eutherians, it is important to recognize that all marsupials have a yolk sac placenta and that, to some degree, the koala, wombats, bandicoots, and certain dasyurids have a form of chorioallantoic placentation. The reproductive strategy of marsupials is different, of course, with a highly altricial neonate undergoing much of its development in the pouch ^16^. There is physiologically significant overlap in gene expression among the eutherian placenta, marsupial yolk sac, and marsupial mammary gland ^17^. Comparative transcriptomics can even explore patterns of gene expression common to mammals and viviparous reptiles and fish ^18– 20^. Because convergent evolution of placentation has occurred many times in vertebrates ^21^, the findings bear on the wider issue of how new organs and new functions have evolved; for other major organs, this would entail comparison between whole phyla and a much deeper timescale ^22^.

## Endogenous retroviral genes and the placenta

Human placental villi are clothed by two layers of trophoblast. The syncytiotrophoblast faces the intervillous space where the maternal blood circulates. The inner layer of cytotrophoblast is continuous in the first trimester, although the cells are more scattered at term ^23^. Syncytiotrophoblast lacks inner cell boundaries but contains multiple nuclei. These undergo apoptosis and the apoptotic nuclei accumulate in syncytial knots that are shed into the maternal bloodstream ^24, 25^. Syncytiotrophoblast therefore requires continual replenishment, and that is achieved by fusion with the cytotrophoblasts ^26^. This process requires the expression of syncytins, which are coded by endogenous retroviral envelope ( *env*) genes. In viruses, the *env* gene product facilitates fusion of the viral membrane with the plasma membrane of the host cell. It also has immunosuppressive properties. Capture of retroviral *env* genes has occurred multiple times during the evolution of mammals and is seen to be essential for the formation of syncytial trophoblast ^27^. Indeed, it has been suggested that retroviral gene capture was pivotal for the evolution of placentation ^27^. An extension of this hypothesis embraces the sushi–ichi-related retrotransposon homolog family, an example being *Peg10*, which is required for placental development in rodents ^28^. This imprinted gene is conserved in eutherians and marsupials ^29^ and has a sequence corresponding to the *gag* and *pol* regions of retroviral genomes though without the *env* region.

Two human syncytin genes are known and there are likewise two syncytins in the mouse. Because each of these represents a separate gene capture, it was pertinent to search for similar genes in other mammals. To fulfil the requirements for a syncytin, the gene had to be able to promote cell fusion in an appropriate assay and the gene or its product shown to be expressed in placental tissue. To date, syncytin genes have been verified in the woodchuck (a squirrel-like rodent) ^30^, guinea pig ^31^, mole rat ^32, 33^, rabbit ^34^, hedgehog tenrec ^35^, and pecoran ruminants ^36, 37^. The basic structure of the ruminant placenta is epitheliochorial. Yet the binucleate trophoblast cells of ruminants are able to fuse with uterine epithelial cells to form heterologous trinucleate cells (in cattle) or more extensive syncytial plaques (in sheep) ^38^. The finding that a syncytin was expressed in binucleate cells was of interest, since it provided a mechanism for one of the enigmas of mammalian placentation. Syncytiotrophoblast also occurs in a few marsupials, where it is associated with a yolk sac placenta. Therefore, it is significant that a syncytin could be demonstrated in the gray short-tailed opossum ( *Monodelphis domestica*) ^39^. Finally, endogenous retroviral *env* genes have been documented in a lizard ( *Mabuya* spp.); one of them has the properties of a syncytin and is expressed in the placenta at the fetal–maternal junction, including in a maternal syncytial layer ^40^.

## Transcriptome of the eutherian placenta

Many genes are involved in placental development and function. There are, however, rather few placenta-specific genes. Moreover, those identified seem not to be expressed in all types of placenta ^41^. An example is trophoblast-specific protein ( *Tpbp*) expressed in the mouse during formation of the placenta and in the spongiotrophoblast of the mature organ. Orthologous genes are found only in rodents ^41^. Placenta-specific genes often arise through gene duplication. Thus, placental lactogens have evolved convergently in primates, rodents, and ruminants through duplication of the growth hormone and prolactin genes ^14, 42^. In the absence of placenta-specific genes, it follows that placental development is organized by the genes that direct similar processes in other organs. Starting with a classic set of gene knockouts in the insulin-like growth factor system ^43^, genes essential to placental development in the mouse were progressively catalogued on the basis of mouse mutants ^44^. But those genes may not be important for all types of eutherian placentation ^45^.

The risk inherent in relying on the mouse as a model for human placenta, let alone other types of placentation, has been underscored by transcriptomics. Even a summary comparison of placental transcriptomes from mouse and human placenta revealed important differences ^46^. A significant first step toward a broader approach involved comparing the transcriptome of elephant placenta with that of mice, humans, and cows ^45^. In an insightful analysis, the authors looked for a subset of 245 genes known to give abnormal placental morphology when knocked out in the mouse. Only 90 of those genes were expressed in all four placental types examined, although an additional 62 were shared by mice and humans. Until recently, data for other mammals were limited. This was remedied in a study that compared transcriptomes from term placentas of 14 species from seven orders representing most major branches in the mammalian tree ^47^. The authors were able to define a set of 115 core genes that were highly expressed in all eutherian placentas. These comprised genes implicated in immune tolerance and cell–cell or cell–matrix interactions. Of interest, they included four components of annexin complexes, consistent with a putative role for annexins in immune regulation ^48^. Another annexin in the set ( *ANXA5*) is thought to promote cell fusion and may be important for syncytiotrophoblast formation and membrane repair ^49^. There was also high expression of key components of the epidermal growth factor receptor (EGFR) signaling pathway, which is associated with the invasive properties of trophoblast ^50^. One limitation of the study was that data were not available from early pregnancy, when genes involved in the development of placental form might be more highly expressed. This could explain why it was not possible to identify genes that correlated with differences in placental interdigitation or invasiveness. Nevertheless, this article strengthened the case that transcriptomics may be key to understanding how the placenta has evolved.

The world was peopled following the migration of anatomically modern humans from Africa some 200,000 years ago ^51, 52^. Not only did humans continue to evolve but also new genes were acquired through interbreeding with other hominins, both within ^53, 54^ and outside ^51^ Africa. An example is introgression of Neanderthal HLA haplotypes of import for immune function ^55^. Another is the diminished erythropoietic response of Tibetans to low ambient oxygen, which is due to introgression of a Denisovan variant of the gene ( *EPAS1*) that encodes hypoxia-inducible factor 2α ^56^. It is to be anticipated that evolutionary changes will be reflected in placental transcriptomes. A step in the right direction is a study comparing the transcriptomes of Americans of different ancestry (Europe, Africa, South Asia, and East Asia) ^57^. Significant differences among groups emerged in pathways related to immune responses, cell signaling, tissue development, and metabolism. There was evidence for adaptive responses in non-African populations following migration out of Africa. However, the African-American population of this study was not well characterized. Since Africa harbors greater genetic diversity than the rest of the world ^54^, it would be informative to see a population-based study of placental transcriptomes from that continent. Furthermore, it might be useful to examine placental transcriptomes in human populations residing at high altitude, as they exhibit many adaptations in placental structure and function to counter chronic hypoxia ^58– 60^.

Most work on placental transcriptomes has been concerned with delivered placentas and focused on the fetal part of the placenta. Fetal membranes have usually been discarded, and since much of the maternal contribution to the placenta has been retained, it is unavailable for study.

## Transcriptome of endometrium and decidua

In preparation for pregnancy, the endometrium undergoes a process called decidualization. This involves a change in the size, shape, and properties of the connective tissue cells (stromal fibroblasts). Decidualization is a prerequisite for implantation of the blastocyst and often occurs in response to an embryonic signal. This does not occur in marsupials ^61^. Based on a phylogenetic analysis, we concluded that decidualization was present in the MRCA of eutherians but was lost in some lineages, especially those that evolved non-invasive epitheliochorial placentation ^4, 62^.

Transcriptomic analysis has thrown new light on the evolution of the decidualization process ^63, 64^. Kin *et al*. ^64^ compared the transcriptomes of endometrial stromal cells in a marsupial, the gray short-tailed opossum, and five eutherians: human, rat, rabbit, and mink, where a decidual reaction occurs, and the cow, where it has been lost. Eutherian expression patterns differed from those of the marsupial in interesting ways, including the loss of genes associated with the immune response and inflammation and the recruitment of *FOXM1*, a gene expressed during the decidualization process. Noting that marsupial gestation seldom exceeds the length of a sterile sexual cycle, whereas eutherian gestation invariably does, these results were leveraged to further the idea that the evolution of the decidual reaction was a critical step toward the eutherian mode of reproduction ^64, 65^. Published data on the decidual reaction and novel observations were marshalled in support of this hypothesis ^65^. Importantly, it could be shown that the decidual reaction is a transient response in many species and is associated mainly with embryo implantation. In a subset of mammals, decidual cells persist and play an additional role in pregnancy maintenance. This happens in humans and many other primates, and this new function possibly emerged in the MRCA of Euarchontoglires, a clade that includes primates, rodents, lagomorphs, tree shrews, and colugos ^65^. These insights should lessen confusion about the relationship between the decidual reaction and the concept of “deciduate” placentation ^66^, where endometrial components are shed with the placenta at birth. Even Mossman ^67^, who was at pains to distinguish between the two, wrote of “atypical decidua” in elephants and carnivores. That such a view no longer is tenable is a good illustration of the power of transcriptomics to address fundamental biology.

In an extension of this work, transcriptomic data were used to explore differences and similarities between the decidual response and the inflammatory response ^68, 69^. It was shown for the opossum that the brief period of attachment between fetal membranes and the uterus was characterized by expression of the full repertoire of genes characterizing the inflammatory response ^63, 69^. In eutherians, on the other hand, some components were missing. These included genes associated with the recruitment of neutrophils ( *CXCL8* and *IL17A*), an immune response that would prove detrimental to an implanting embryo. There was also downregulation of prostaglandin (PG) F _2α_ synthesis. Expression of many other components was retained and beneficial to implantation. These included genes promoting vascular permeability through PGE _2_ signaling, activation of regulatory immune cells, and production of acute-phase proteins. As the authors acknowledged ^68, 69^, the validity of this hypothesis needs to be tested with endometrial transcriptomes from mammals at the basal nodes of the eutherian tree, including Afrotheria (for example, elephants and tenrecs) and Xenarthra (for example, armadillos and sloths).

## Transcriptome of marsupial yolk sac and mammary gland

In marsupials, gestation is supported by a yolk sac placenta. There is evidence for a division of function between distinct areas of the yolk sac. The non-vascular part (bilaminar omphalopleure) is responsible for the uptake and metabolism of nutrients, and the vascular part (trilaminar omphalopleure) is important for respiration. A recent article compared the transcriptomes of marsupial (tammar wallaby, *Macropus eugenii*) and eutherian (mouse and human) placentas and mammary glands ^17^. It confirmed that marsupials have fully functional placentas expressing many of the same genes as eutherian ones. A fascinating detail was that the yolk sac endoderm of the tammar had assumed functions that in eutherians are served by trophoblast; these had to do with the trafficking of nutrients. Because much of the development in the wallaby is supported by lactation, it was interesting to find considerable overlap in the transcriptomes of the marsupial mammary gland and eutherian placenta. Several eutherians, including the mouse, have a yolk sac that supports early embryonic development and continues to function alongside the chorioallantoic placenta until term ^9^. Perhaps owing to unavailability of a mouse yolk sac transcriptome, this was not included in the comparison.

## Transcriptome of the yolk sac in humans, mice, and chickens

Some mammals, including higher primates, have a yolk sac that does not make contact with uterine tissues and therefore is not regarded as a placenta ^9^. A case in point is the secondary yolk sac found in the first trimester of human pregnancy. Apart from an acknowledged role in hematopoiesis, it is widely regarded as a vestigial organ. This view needs revision in light of a recent study of its transcriptome ^18^. The results justify the conclusion that the placenta, exocelomic fluid, and yolk sac together represent an important route for maternal–fetal transfer early in human gestation. Thus, the most highly expressed genes included those for the solute carrier (SLC) transporters as well as ABC transporters that transport cholesterol and lipids and facilitate the excretion of toxins. As expected, the role of the yolk sac in hematopoiesis was reflected in the expression of associated genes, including that for the β-chain of embryonic hemoglobin ( *HBZ*).

The same study had a comparative aspect, contrasting the transcriptomes of chicken, mouse, and human yolk sacs ^18^. While this provided evidence for conservation of function, analysis of the mouse yolk sac transcriptome was not as thorough as might be desired and mouse placenta was not included.

## Viviparity and placentation in other vertebrates

Live birth, often in association with placentation, occurs in every class of vertebrate (though not in birds) ^21^ as well as in tunicates ^70^ and invertebrates ^71^. However, only amniotes have the full complement of fetal membranes familiar from mammals. A recent study ^19^ compared transcriptomes from the chorioallantoic membranes of the chicken, oviparous and viviparous lizards, and the horse. Focusing on hormone-related genes, they found 91 that were expressed in all four species. These included genes coding for enzymes involved in cholesterol and steroid synthesis; insulin-like growth factor II ( *IGF2*) and its receptor ( *IGF1R*); and genes associated with thyroid hormone receptor binding. It was concluded that the chorioallantoic membrane had an endocrine function in the MRCA of amniotes that was subsumed in those with chorioallantoic placentation. Interestingly, no gene in their data set was expressed exclusively by the two viviparous species.

Another study on the evolution of placentation in lizards focused on the Australian southern grass skink ( *Pseudemoia entrecasteauxii*), which has both yolk sac and chorioallantoic placentation ^72^. Gene ontology analysis reinforced the view that the two sets of fetal membranes support different functions. Thus, chorioallantoic membranes were enriched in SLC transporters, suggesting that they are the primary site of solute transport, while the yolk sac expressed genes associated with lipid transport in vesicles. Moreover, identification of the chorioallantoic placenta as the primary site of respiratory gas exchange was consistent with increased uterine expression of vascular endothelial growth factor at this site. In contrast, no strong differences in gene expression occurred between gravid and non-gravid uteri in two species of oviparous skink.

Viviparity in seahorses and pipefish involves incubation of the embryos in the brood pouch of the male. Recently, Whittington *et al*. ^20^ analyzed the transcriptome of the brood pouch of the pot-bellied seahorse ( *Hippocampus abdominalis*) for genes that are upregulated during pregnancy and in transition to the postpartum state. Genes upregulated in pregnancy were associated with tissue remodeling, nutrient transport (for example, the SLC family), and immune regulation. Many of the same genes or their homologs are upregulated during pregnancy in mammals and other viviparous taxa. Genes downregulated in the pregnant brood pouch included some associated with the inflammatory response. The authors suggest the possibility that a common toolkit of genes is recruited to support pregnancy in mammals, reptiles, and live-bearing fish.

## Concluding remarks

Transcriptomics is a powerful tool for understanding placentation and its evolution in eutherian mammals. It is important that future studies cover all major groups, including the basal Afrotheria and Xenarthra. Whereas studies hitherto have focused on the placenta at term, it is important to know what genes are expressed during the early development of the placenta. Even findings from a more limited set of mammals would be welcome in that respect. Mammals present an impressive degree of variation not only in the chorioallantoic placenta but also in the fate of the yolk sac and in paraplacental structures ^73^. These should not go unheeded. It is not known, for example, how gene expression is allocated between the inverted yolk sac and chorioallantoic placenta of the mouse and how these together compare with human placenta.

Single-cell transcriptomics has furthered understanding of embryonic development in mice and humans. Studies have appeared on lineage commitment in mouse embryonic stem cells ^74^ as well as on embryonic and trophoblast fate specification in blastomeres from 8- to 12-cell human embryos ^75^. However, these findings may not translate to other species. It is, for example, known that *Oct4* expression is repressed by *Cdx2* in trophectoderm of the mouse blastocyst but not in the bovine blastocyst ^76^, whereas in the tammar wallaby, *CDX2* seems to play no role in the differentiation of the unilaminar blastocyst into trophoblast and pluriblast (the forerunner of epiblast and hypoblast) ^77^. Later in development, the placenta has multiple cell types of both fetal and maternal origin. Single-cell transcriptomics should enable the interplay between cell types to be uncovered as already attempted for human placenta at term ^78^. The cells comprising a placenta vary greatly across species. Thus, both early in embryonic development and in relation to the mature placenta, single-cell transcriptomics has the potential to enhance our knowledge of how the placenta has evolved. The sheer diversity of placentation has fascinated scientists since the 19th century. Transcriptomics has the potential to help explain it in functional terms.
